# Use of Wild Bird Surveillance, Human Case Data and GIS Spatial Analysis for Predicting Spatial Distributions of West Nile Virus in Greece

**DOI:** 10.1371/journal.pone.0096935

**Published:** 2014-05-07

**Authors:** George Valiakos, Konstantinos Papaspyropoulos, Alexios Giannakopoulos, Periklis Birtsas, Sotirios Tsiodras, Michael R. Hutchings, Vassiliki Spyrou, Danai Pervanidou, Labrini V. Athanasiou, Nikolaos Papadopoulos, Constantina Tsokana, Agoritsa Baka, Katerina Manolakou, Dimitrios Chatzopoulos, Marc Artois, Lisa Yon, Duncan Hannant, Liljana Petrovska, Christos Hadjichristodoulou, Charalambos Billinis

**Affiliations:** 1 Faculty of Veterinary Medicine, University of Thessaly, Karditsa, Greece; 2 Department of Biomedicine, Institute for Research and Technology of Thessaly, Centre For Research and Technology Hellas, Larissa, Greece; 3 Research Division, Hunting Federation of Macedonia and Thrace, Thessaloniki, Greece; 4 Technological Education Institute of Thessaly, Larissa, Greece; 5 Hellenic Center for Disease Control and Prevention (HCDCP) Ministry of Health, Athens, Greece; 6 National and Kapodistrian University of Athens, Faculty of Medicine, Athens, Greece; 7 Disease Systems, SRUC, Edinburgh, United Kingdom; 8 Department of Agriculture Crop Production and Rural Environment, University of Thessaly, Volos, Greece; 9 University of Lyon, VetAgro Sup, France; 10 School of Veterinary Medicine & Science, University of Nottingham, Nottingham, United Kingdom; 11 Animal Health and Veterinary Laboratories Agency - Weybridge, United Kingdom; 12 Faculty of Medicine, University of Thessaly, Larissa, Greece; Centers for Disease Control and Prevention, United States of America

## Abstract

West Nile Virus (WNV) is the causative agent of a vector-borne, zoonotic disease with a worldwide distribution. Recent expansion and introduction of WNV into new areas, including southern Europe, has been associated with severe disease in humans and equids, and has increased concerns regarding the need to prevent and control future WNV outbreaks. Since 2010, 524 confirmed human cases of the disease have been reported in Greece with greater than 10% mortality. Infected mosquitoes, wild birds, equids, and chickens have been detected and associated with human disease. The aim of our study was to establish a monitoring system with wild birds and reported human cases data using Geographical Information System (GIS). Potential distribution of WNV was modelled by combining wild bird serological surveillance data with environmental factors (e.g. elevation, slope, land use, vegetation density, temperature, precipitation indices, and population density). Local factors including areas of low altitude and proximity to water were important predictors of appearance of both human and wild bird cases (Odds Ratio = 1,001 95%CI = 0,723–1,386). Using GIS analysis, the identified risk factors were applied across Greece identifying the northern part of Greece (Macedonia, Thrace) western Greece and a number of Greek islands as being at highest risk of future outbreaks. The results of the analysis were evaluated and confirmed using the 161 reported human cases of the 2012 outbreak predicting correctly (Odds = 130/31 = 4,194 95%CI = 2,841–6,189) and more areas were identified for potential dispersion in the following years. Our approach verified that WNV risk can be modelled in a fast cost-effective way indicating high risk areas where prevention measures should be implemented in order to reduce the disease incidence.

## Introduction

**Figure 1:**
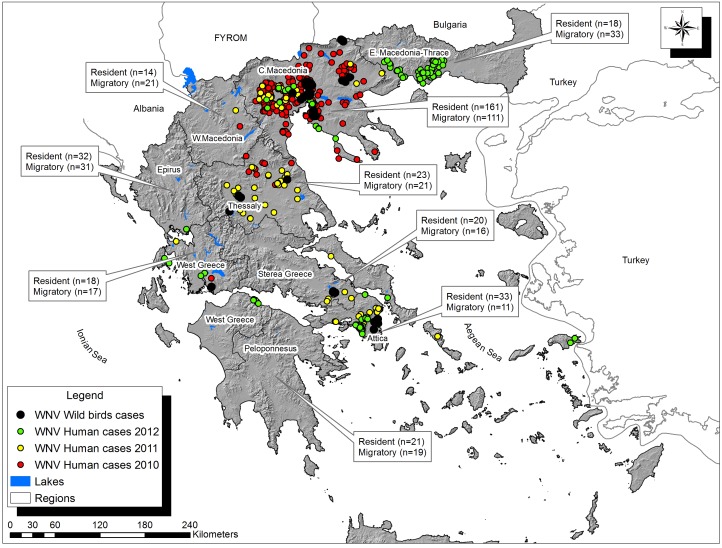
Map of Greece showing WNV laboratory-confirmed human cases and seropositive resident wild birds, 2010–2012. Map of Greece showing the distribution of WNV laboratory-confirmed human cases and seropositive resident wild bird samples for the 2010–2012 period. Red, yellow and green dots indicate human cases reported in 2010, 2011 and 2012 respectively. Black dots indicate seropositive resident wild birds detected during the same period. Text boxes refer to available avian samples (resident and migratory) per each region.

West Nile virus (WNV) is a mosquito-borne flavivirus with increasing numbers of reported human disease cases worldwide. In Europe, cases of WNV associated diseases have been reported in several countries in the European Union and in bordering Non-E.U. countries. The largest ongoing European outbreak has been observed in Greece, with more than 524 confirmed cases of human infection and 60 deaths reported since 2010 [1] (Figure 1).

Many studies have associated the presence of specific environmental factors with areas at high-risk for WNV transmission in the USA [2]–[5] and Europe [6], [7]. Tachiiri et al. (2006) developed a model using basic geographic and temperature data to assess WNV risk in British Columbia [8]. Ruiz et al. (2004) used several factors related to the physical environment such as elevation range, physiographic region, and percentage of vegetation cover to determine WNV risk during an outbreak in the Chicago area in 2002 [9]. Methods that have been used in WNV risk modeling include non-linear discriminant analysis [10], logistic [11] or multiple regression models [12] (differential and difference equation modeling [13], [14] and cluster analysis [15].

Predictive modeling with Geographic Information System (GIS) can be used to analyze environmental determinants of WNV transmission and determine high risk areas. Most previous WNV risk analyses utilized spatial statistical techniques (mapping clusters, geographic distribution, spatial relationships-regression models) to correlate environmental, climatic and socioeconomic factors with WNV prevalence [2], [4], [5], [9], [11], [16].

The geographical position of Greece in the Mediterranean peninsula makes it an important transit zone for migratory birds [17]. Greece hosts a wealth of biological diversity, one of the richest in Europe and the Mediterranean.

The main objective of this study was to correlate serological data of exposure of wild birds to WNV and reported human cases data during the Greek outbreak with potential environmental risk factors within a GIS, in order to construct predictive maps identifying areas at risk from further spread. We test the predictive power of the models against recent outbreak data and identify high risk areas for the application of targeted, timely and cost-effective prevention measures such as surveillance, mosquitoes control and campaigns to increase public awareness of the disease.

## Materials and Methods

### Ethics Statement

This study was co-funded by the «Integrated Surveillance and control programme for West Nile Virus and malaria in Greece» (National Strategic Reference Framework, 2007–2013) and was also part of European Union Seventh Framework Programme (2007–2013) large collaboration project under grant agreement no. 222633 (Novel Technologies for Surveillance of Emerging and Re-emerging Infections of Wildlife - WildTech). University of Nottingham is the project coordinator, and has received signed cooperation agreements by all the project partners, for providing animal samples to the project. All samples used in this project represent material collected by partners and other organisations for other purposes than this project as specified in deliverable D4.5/5.5 entitled “Guidelines for ethical sample collection” submitted to European Commission (26/02/2010, Dissemination Level: PP, Restricted to other programme participants, including Commission Services). The avian samples were collected opportunistically (no active capture, killing and sampling of wild animals specifically for this study was performed) from animals hunter-harvested by members of Greek Hunting Federation of Macedonia and Thrace, from species considered quarry and during the hunting seasons, according to the prerequisites of the Greek Legislation (FEK 1611/B′/2009, FEK 1183/6-8-2010, FEK 1763/4-8-2011), thus special approval was not necessary and steps to ameliorate suffering were not applicable in this study. Research on animals as defined in the EU Ethics for Researchers document (European Commission, 2007, Ethics for Researchers - Facilitating Research Excellence in FP7, Luxembourg: Office for Official Publications of the European Communities, ISBN 978-92-79-05474-7) is not a part of the study.

The human data were part of the ongoing surveillance of human cases performed by the Hellenic Center for Disease Control and Prevention (HCDCP) and were reported from the treating physicians. Data on human cases is maintained on a database kept by the HCDCP that was completely anonymized to the authors, without being publicly available. Since residential addresses could be potentially identifying, the security of this database was maintained according to the national regulation for the confidentiality of human data. The use of the residential addresses has been approved by the Institutional Review Board (IRB) of the Public Health and Environmental Hygiene post graduate course of the Laboratory of Hygiene and Epidemiology, Faculty of Medicine, University of Thessaly, Greece. The IRB waived the need for written informed consent to use the residential addresses, with the restriction that the analysis would be carried out at the municipality level.

### Study Area

The study area comprised the entire country of Greece. Greece occupies the southeastern part of Europe with a total area of 131,990 km^2^. Eighty percent of Greece consists of mountains; the country is characterized by a large climatic diversity (29 climatic zones according to the Thorn Waite classification), by its extensive coastline of about 15,000 km and many island complexes in the Archipelagos of Aegean Sea and the Ionian Sea. Climatic conditions of the country are typical Mediterranean: Summer is hot and dry while winter is usually mild. Rain mostly falls in autumn and winter.

### WNV Human Cases Data

Reported human WNV cases in Greece (2010–2012) were provided by HCDCP. Most cases were serologically confirmed by the presence of IgM antibodies in the serum and/or the cerebrospinal fluid. Residential address of each human case was used for geocoding and mapping the cases.

### Wild Birds Surveillance

A total of 620 avian serum samples were obtained from wild birds hunter-harvested by members of the Greek Hunting Federation of Macedonia and Thrace, from species considered quarry during the 2009/2010, 2010/2011 and 2011/2012 hunting seasons (from 20 August until 28 February the following year), according to the prerequisites of the Greek Legislation. All available samples were obtained from mainland Greece, opportunistically collected during regular hunting activities; samples were available from all 9 mainland regions of Greece (Table 1). Sampling effort was distributed in mainland Greece, avoiding cluster sampling biases, with the exception of the Central Macedonia region, the epicenter of the outbreak, during which a large number of samples were provided. Data on bird specimens that tested positive for WNV during the study were located in the field using handheld Global Positioning System (GPS) units or located by means of longitude and latitude information provided by samplers. Serological screening was performed as already reported [18]–[20]; a total of 64 resident wild birds were found positive for WNV antibodies, and were used in the current study (migratory wild birds were also found seropositive, but relevant data was excluded from the analysis, see Discussion).

**Table 1:** Available avian samples: species, migratory status and number of samples per region.

Species	Migratory Status	E. Macedonia & Thrace	Central Macedonia	Thessaly	Epirus	West Greece	Peloponnesus	Sterea Greece	Attica	West Macedonia
*Pica pica*	Resident	11	125	17	20	12	14	13	29	9
*Streptopelia turtur*	Migratory	8	35	4	11	7	8	7	5	8
*Corvus cornix*	Resident	4	18	5	8	4	4	6	4	3
*Anas platyrhynchos*	Migratory	5	26	3	0	0	0	2	0	0
*Scolopax rusticola*	Migratory	4	12	5	9	3	6	2	0	5
*Turdus philomelos*	Migratory	7	27	6	8	7	5	4	6	8
*Corvus monendula*	Resident	3	18	1	4	2	3	1	0	2
*Anas crecca*	Migratory	9	11	3	3	0	0	1	0	0
Total		51	272	44	63	35	40	36	44	35

### Environmental Variables

Environmental variables for this study were derived from three main database categories: climate, elevation and land cover data.

WorldClim version 1.4 climate data [21] was obtained from the WorldClim website (http://www.worldclim.org). WorldClim is a set of global climate layers (climate grids) with a spatial resolution of 1 square kilometer. Topographic variables including altitude, aspect and slope were extracted from a digital elevation model (DEM) with a spatial resolution of 1 square kilometer (http://srtm.csi.cgiar.org/Index.asp). Land uses were derived from the Corine Land Cover 2000 database (European Environment Agency – EEA, http://www.eea.europa.eu/data-and-maps).

Village and vegetation corrections were digitized from 2007 and 2009 color orthophotos that were available through Web Mapping Service (WMS) (http://gis.ktimanet.gr). To create environmental layers (n = 37) for the analysis (Table 2), ArcGIS 10.1 GIS software (ESRI, Redlands, CA, USA) was used. GIS layers were created to represent factors like the locations of towns and villages, distance to the nearest village, distance from water presence etc. For many of the above parameters, we calculated neighborhood statistics for radii of 100, 200, 500 and 1000 m to determine which spatial scale affects the presence of cases most strongly. These data sets were converted to a common projection, map extent and resolution prior to use in the modeling program.

**Table 2:** Environmental variables used in the analysis.

Variable	Value	Source
Slope (degrees) at 100-, 200-, 500- and 1000-m radii	X, SD, min, max	DEM
Topographic position index (4 classes)	binary	DEM
Altitude	continuous	DEM
Aspect	N,W,S,E	DEM
Distance from nearest village (m)	continuous	ArcGIS-WMS
Distance from water (m)	continuous	ArcGIS-DEM
Habitat types (4 classes: Forests, cultivations, etc.)	Categorical transform to continuous	ArcGIS-Corine LC (EEA)
19 Climatic variables (Temperature 11 indices, Precipitation 8 indices)	continuous	World Clim Database
NDVI (Normalized Difference Vegetation Index) 12 indices	continuous	World Clim Database
Population density	continuous	GEoDatabase

### Statistical Analysis

We used data on 2010 and 2011 human cases for the statistical analysis and model building and kept the 2012 cases for verification. A total of 363 human WNV cases have been reported in Greece for the years 2010 and 2011 (262 cases in 2010 and 101 cases in 2011). The available dataset consisted of presence only data (presence: people infected by the virus). For this dataset, as well as the wild birds seroprevalence dataset a number of explanatory variables (n = 37) were collected and constructed, as mentioned previously (Table 2).

Instead of constructing a number of pseudo-absence controls, a methodology which according to the literature has some significant disadvantages for the prediction modeling [22], [23], we decided to search for within the presence data variation of the explanatory variables. We clustered the cases using the agglomerative method of Two Step Cluster Analysis, a method which allows for the utilization of both continuous and categorical variables and clusters the cases by measuring the log-likelihood distance among them [24]. The Two Step Cluster Analysis allowed us to check for a pattern of the virus among the infected people in 2010, in 2011, and in total. The optimal number of clusters was chosen using the Silhouette coefficient, a measure proposed by Kaufman and Rousseeuw (1990) [25]. The coefficient ranges from −1 to 1 and when its value is closer to 1, the clustering is considered efficient.

Before applying the above cluster method, we checked a number of descriptive statistical measures which describe our data. Although Two Step Cluster Analysis is robust to non-normality [24] we used Factor Analysis in order to reduce the number of available variables and to achieve normality and zero-correlation among explanatory continuous variables. We used the Principal Component Analysis (PCA) as a method of components extraction with rotation method the Varimax method with Kaiser Normalization [26]. Two Step Cluster Analysis was iterated several times using as clustering variables either the components which were extracted by the Principal Component Analysis, or the original variables which were highly correlated with the components. The extracted clusters for humans and the extracted clusters for birds were compared in terms of the variables that are important for clustering.

### GIS Analysis

Two significant environmental variables were recognized from the statistical analysis (see Results) and were used to measure environmental conditions for the WNV locations of the seropositive wild birds and the human cases dataset. Mahalanobis distance (MD) [27] was used to develop a distance measure model for wild birds and predict WNV potential distribution prior to the expansion/outbreak of the 2012 period. We calculated MD with ArcGIS software, based on the values of the two significant variables, allowing us to identify suitable areas for WNV potential distribution and occurrence. Model performance evaluation was conducted with the 2012 reported WNV human cases, as provided by HCDCP.

## Results

Data analysis demonstrates differences between 2010 and 2011 in terms of positive cases in Greece (Table 3). Fewer cases (n = 101) occurred in 2011 and the average case age was 5 years younger compared to 2010 (p-value = 0.024). There was a statistically different distribution in terms of the prefecture of residency of the positive cases, which were found in more southern areas compared to 2010, indicating the pathogen’s continued spread in mainland Greece.

**Table 3:** Descriptive statistics of positive human cases 2010–2011.

Variable	2010	2011	Total
Positive cases	262	101	363
Age*	67.5 (sd = 16.9)	62.4 (sd = 19.9)	66 (sd = 17.9)
Sex	47.7% women	36.6% women	44.6% women
Prefecture of residency+	31% Thessaloniki	30% Attiki	25% Thessaloniki
	22% Pella	15% Larissa	18% Pella
	19% Imathia	10% Thessaloniki	15% Imathia
Date of infection	11.8% July	20.8% July	
	63.8% August	45.5% August	
	24.0% September	30.7% September	
	0.4% October	3% October	

*Statistically significant reduction in 2011 (5 years) (p-value = 0.024<0.05).

+ *Independence between 2010 and 2011 (p-value = 0.000<0.001).*

Finally, the distribution of the infected individuals in terms of date of infection was different in 2011, where more positive cases were found in July and September, compared to 2010 where the majority of the cases were found in August (64%).

**Figure 2:**
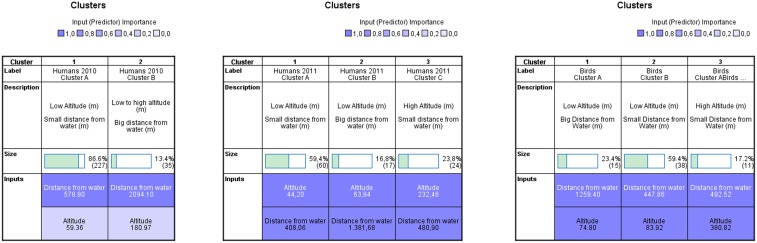
Clusters of human cases of seropositive wild birds and reported human cases of 2010–2011. Clusters of WNV reported human cases of 2010–2011 and seropositive wild birds, according to attributes of altitude and distance from water. Mean values of the two variables are presented under each cluster.

Factor Analysis and Two Step Cluster Analysis revealed that altitude and distance from water were the two variables, among the 37 under study, which clustered significantly the cases. Both variables played a significant role in the clustering procedure. The two variables clustered in a similar way for both humans and birds. For the clustering of human cases, the average Silhouette coefficient was 0.5 which is considered a good clustering value [25]. The same value was achieved for the clustering of seropositive wild birds (Odds Ratio = 1,001 95%CI = 0,723–1,386). Three clusters were created for humans and birds (Figure 2), sharing the same attributes. In particular, humans’ Cluster A and birds’ Cluster B share the majority of the positive cases in humans and birds respectively (60%). There seems to be a pattern of WNV in Greece in places with low altitude and small distance from water. There are also two other clusters with lower percentages of cases which show that positive cases are also found in places with low altitude and big distance from water (23–24%) and in places with high altitude and small distance from water (almost 17%).

**Figure 3:**
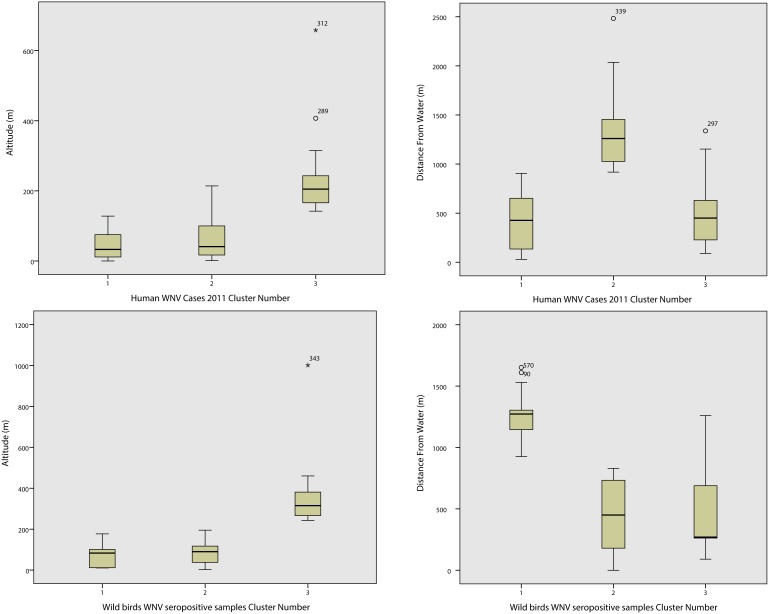
Box-plots of range and altitude. Box-plots displaying range of altitude (left) and distance from water (right) in the three clusters of humans 2011 WNV positive cases and seropositive wild birds.

Relevant box-plots (Figure 3) show how well the two variables discriminate in each cluster. A clear separation of the three clusters is seen in both groups of cases.

Regarding the 2010 human cases, clustering showed that low altitude and small distance from water were associated with the majority of the positive human cases as well. A total of 86.6% of the human cases were grouped in cluster A (Figure 2), which shares similar attributes with cluster A of human 2011 cases and cluster B of birds.

**Figure 4:**
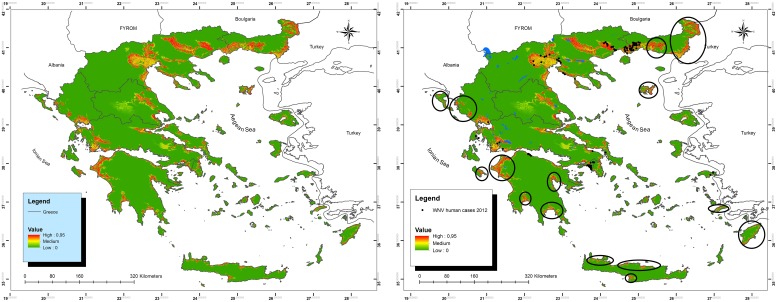
Map of Greece showing potential geographic distribution of WNV. Map of Greece showing potential geographic distribution of WNV, predicted by GIS and MD based on the attributes of the major clusters of reported human cases of 2011 and seropositive wild birds (low altitude, small distance from water). Black dots indicate reported human cases in 2012. Black circles indicate suggested high-risk areas for WNV further dispersion in following years.

The potential geographic distribution of WNV, predicted by GIS and MD based on the attributes of the major clusters of reported human cases of 2011 and seropositive wild birds is displayed in Figure 4. Fragmented high-risk areas were recognized: Most were concentrated in the Macedonian prefecture, in western Greece as well as in Thessaly. Other suitable high risk areas were located along the coast line of the Peloponnese peninsula and Crete. Moreover, many Greek islands have suitable environmental characteristics such as Rhodes, Mytilene, Chios, Samos etc.

In the early transmission period (June 2012) we reported the high–risk areas recognised throughout this study to the Ministry of Public Health and to HCDCP. As already reported, in 2012, a total of 161 laboratory-confirmed human cases were reported. Out of these 161 cases, only 31 occurred far from WNV high-risk areas recognised by our model (Odds = 130/31 = 4,194 95%CI = 2,841–6,189); four (4) human cases out of 5 were reported in recognised high-risk areas while only 1 out of 5 was not. New areas of potential dispersion of the virus are also suggested for the following years in the areas of Thrace, the Peloponnese peninsula and several Greek Islands (Figure 4).

## Discussion

Humans and other mammals, particularly horses, are alternative hosts for WNV; the main route of infection is through the bite of an infected mosquito. Most human infections remain asymptomatic with WNV fever developing in approximately 20% of infected people and West Nile neuroinvasive disease in <1% [28]. Horses and humans develop low viremic loads (<10^5^ PFU/ml) of short duration and thus are considered dead-end hosts for WNV [29]. In contrast, various migratory and resident avian species develop high viremic loads, sufficient to infect feeding ornithophilic mosquitoes [30]. Hence, the WNV life cycle is maintained with birds being the main amplifying hosts and mosquitoes the main vectors. Moreover, local movements of resident birds and long-range travel of migratory birds may contribute to pathogen dispersion [31], [32]. In southern France, WNV was detected in late summer of 2000 and 2004. Migratory passerines were found with higher prevalence of WNV neutralizing antibodies (7.0%) than resident and short-distance migratory passerines (0.8%), suggesting exposure to WNV or a related flavivirus during overwintering in Africa [33]. Additionally in Spain it was found that Trans-Saharan migrant species had both higher prevalence and antibody titres than resident and short-distance migrants [34].

In Greece, the disease first appeared in Macedonia prefecture in 2010, with 262 confirmed cases and 35 deaths, and it subsequently spread through mainland Greece in the following years. More specifically, in 2011, the outbreak expanded southwards to central Greece with 101 confirmed cases and 9 deaths, while in 2012, a total of 161 confirmed cases and 18 deaths were reported mainly in Attica and northeastern Greece. A strain of lineage 2 was detected in 2010 in pools of Culex mosquitoes [35] and in wild birds [20]. In this study we correlate various environmental factors with WNV maintenance, amplification and potential for future spread in Greece.

Various public health studies have used Geographical Information System technologies as a tool for data analysis [2], [9], [15]. Previous studies [9], [10] found that certain social and environmental factors were correlated with WNV dissemination patterns: The presence of vegetation, distance to a WNV positive dead bird, the intensity of mosquito abatement, demographic factors such as population age, race and financial status. Low precipitation and warm temperature were also found to associate with WNV cases. On the other hand, spread of WNV has shown some unique distribution patterns in different regions [15], [30].

Before reaching the aforementioned results, we undertook several efforts to find out a pattern, or a distinguishable attribute of the WNV positive cases in Greece. Although we used a significant number of explanatory variables for describing each positive case (a mix of both continuous and categorical variables), there was no indication that these altogether could show the pattern in question. Therefore we tried to reduce this dataset by using Factor Analysis. We run PCA once for the temperature variables and once for the precipitation variables. Two components (93% of the variation was explained) for the temperature and two components (96% of the variation) for the precipitation variables were extracted, which means that the fit was very good. These four variables with the rest demographic and environmental variables were used in the Two Step Cluster method. This method was preferred compared to other clustering techniques because it can handle both categorical and continuous variables. However, we also run hierarchical cluster analysis using only the continuous variables, but no pattern of the positive human cases was revealed. Therefore, we used the two step cluster technique in a backward selection way. Initially we used all the explanatory variables together, and we removed one at a time if the Silhouette indicator was not considered good. We used the log-likelihood distance instead of Euclidean distance, because there were initially categorical variables in the dataset. However, when only the two continuous variables were left (“altitude” and “distance from water”), we checked also if the Euclidean distance could reveal the same pattern with the log-likelihood but it didn’t. We believe the fact that some of the variables were not significant for the clustering procedure was due to similar environmental conditions existing in Greece during summer. For example, there is no significant variation in terms of temperature or precipitation. This is why more stable variables like distance from water and altitude were responsible for the form of the clusters. After we formed the three clusters with these two variables, this pattern was revealed for both human cases and resident wild birds seroprevalence data.

Distance to water and altitude have both been previously shown to be negatively correlated with mosquito larval presence [36]; mosquitoes are the main biological vectors of WNV and transmission of this arthropod-borne virus is highly dependent on the density of mosquitoes. Low lying areas in close proximity to water include wetland habitats that are used as resting and breeding areas for various migratory and resident birds, allowing the long-distance introduction of the virus via migration routes as well as the rapid local amplification of the virus in a mosquito-bird cycle. In this study, apart from statistically identifying proximity to water and altitude as risk factors of spread of WNV in Greece, we were able to determine specific mean values for these habitat variables that allowed us to predict areas at high-risk for further disease incursion.

WNV positive birds are considered important environmental predictors of WNV human risk and are used in surveillance and risk assessment [2], [9], [37]. Whilst viremic birds are likely to represent the highest risk to humans, the viremic phase is extremely short, restricting data richness and thus statistical power. Hence we focused our analysis on longer lived serological measures of exposure. Moreover, the use of only the resident WNV seropositive wild birds from all hunter-harvested samples available, even though samples from migratory birds were also found positive, increased the reliability of our analysis, avoiding biases regarding area of exposure e.g. migratory birds travel long distances so the origin of exposure is hard to be determined. Hence, this is a good example of a case in which surveillance regarding exposure and other similar biological data derived from nature, regarding a zoonosis, can be used as an indicator for predicting high-risk areas. This fact was confirmed by the good fit that our model showed for the 2012 positive WNV human cases in Greece.

## Conclusions

Modelling results indicated that positive resident wild bird occurrences are correlated with human WNV risk and can facilitate the assessment of environmental variables that contribute to that risk, recognising new high-risk areas where the disease could further spread. Our approach allowed us to create a risk based mapping system to assist and guide WNV disease surveillance, monitoring and control. This risk based approach offers a way to stratify surveillance efforts and resources to improve the efficiency of surveillance for new outbreaks and monitoring existing outbreaks. Furthermore, it could proactively enhance other preventive efforts and educational campaigns for the general public in the not yet “affected” areas. Most importantly, early warning and identification of outbreaks is critical to limiting the animal and human losses to this disease. An active surveillance program undertaken on resident wild birds could be added to active and passive surveillance focused on humans, horses and mosquitoes greatly helping in evaluating and dealing with future outbreaks linked to flaviviruses.

## References

[1] Hellenic Centre for Disease Control and Prevention – HCDCP (2013) West Nile Virus Epidemic in Greece. Greece.

[2] Cooke WH 3rd, Grala K, Wallis RC (2006) Avian GIS models signal human risk for West Nile virus in Mississippi. Int J Health Geogr 5: 36. 16945154 10.1186/1476-072X-5-36 PMC1618835

[3] Gibbs SE, Wimberly MC, Madden M, Masour J, Yabsley MJ, et al (2006) Factors affecting the geographic distribution of West Nile virus in Georgia, USA: 2002–2004. Vector Borne Zoonotic Dis 6: 73–82. 16584329 10.1089/vbz.2006.6.73

[4] Liu H, Weng Q, Gaines D (2008) Spatio-temporal analysis of the relationship between WNV dissemination and environmental variables in Indianapolis, USA. Int J Health Geogr 7: 66. 19094221 10.1186/1476-072X-7-66 PMC2615761

[5] Rochlin I, Turbow D, Gomez F, Ninivaggi DV, Campbell SR (2011) Predictive mapping of human risk for West Nile virus (WNV) based on environmental and socioeconomic factors. PLoS One 6: e23280. 21853103 10.1371/journal.pone.0023280 PMC3154328

[6] Lelli R, Calistri P, Bruno R, Monaco F, Savini G, et al (2012) West Nile transmission in resident birds in Italy. Transbound Emerg Dis 59: 421–428. 22212727 10.1111/j.1865-1682.2011.01287.x

[7] Rodriguez-Prieto V, Martinez-Lopez B, Martinez M, Munoz MJ, Sanchez-Vizcaino JM (2012) Identification of suitable areas for West Nile virus outbreaks in equid populations for application in surveillance plans: the example of the Castile and Leon region of Spain. Epidemiol Infect 140: 1617–1631. 22126826 10.1017/S0950268811002366

[8] Tachiiri K, Klinkenberg B, Mak S, Kazmi J (2006) Predicting outbreaks: a spatial risk assessment of West Nile virus in British Columbia. Int J Health Geogr 5: 21. 16704737 10.1186/1476-072X-5-21 PMC1523258

[9] Ruiz MO, Tedesco C, McTighe TJ, Austin C, Kitron U (2004) Environmental and social determinants of human risk during a West Nile virus outbreak in the greater Chicago area, 2002. Int J Health Geogr 3: 8. 15099399 10.1186/1476-072X-3-8 PMC420251

[10] Liu H, Weng Q (2008) Seasonal variations in the relationship between landscape pattern and land surface temperature in Indianapolis, USA. Environmental Monitoring and Assessment 144: 199–219. 17899413 10.1007/s10661-007-9979-5

[11] Brownstein JS, Rosen H, Purdy D, Miller JR, Merlino M, et al (2002) Spatial analysis of West Nile virus: rapid risk assessment of an introduced vector-borne zoonosis. Vector Borne Zoonotic Dis 2: 157–164. 12737545 10.1089/15303660260613729

[12] Crowder DW, Dykstra EA, Brauner JM, Duffy A, Reed C, et al (2013) West nile virus prevalence across landscapes is mediated by local effects of agriculture on vector and host communities. PLoS One 8: e55006. 23383032 10.1371/journal.pone.0055006 PMC3559328

[13] Bowman C, Gumel AB, van den Driessche P, Wu J, Zhu H (2005) A mathematical model for assessing control strategies against West Nile virus. Bull Math Biol 67: 1107–1133. 15998497 10.1016/j.bulm.2005.01.002

[14] Cruz-Pacheco G, Esteva L, Montano-Hirose JA, Vargas C (2005) Modelling the dynamics of West Nile Virus. Bull Math Biol 67: 1157–1172. 16125762 10.1016/j.bulm.2004.11.008

[15] Ruiz MO, Walker ED, Foster ES, Haramis LD, Kitron UD (2007) Association of West Nile virus illness and urban landscapes in Chicago and Detroit. Int J Health Geogr 6: 10. 17352825 10.1186/1476-072X-6-10 PMC1828048

[16] Konrad SK, Miller SN, Reeves WK, Tietze NS (2009) Spatially explicit West Nile virus risk modeling in Santa Clara County, California. Vector Borne Zoonotic Dis 9: 267–274. 19514810 10.1089/vbz.2008.0084

[17] Handrinos G and Akriotis T (1997) The Birds of Greece. London: C. Helm.

[18] Valiakos G, Touloudi A, Athanasiou L, Giannakopoulos A, Iacovakis C, et al (2012) Exposure of Eurasian magpies and turtle doves to West Nile virus during a major human outbreak, Greece, 2011. European Journal of Wildlife Research 58: 749–753.

[19] Valiakos G, Touloudi A, Athanasiou L, Giannakopoulos A, Iacovakis C, et al (2012) Serological and molecular investigation into the role of wild birds in the epidemiology of West Nile virus in Greece. Virology Journal 9: 266. 23140247 10.1186/1743-422X-9-266 PMC3546012

[20] Valiakos G, Touloudi A, Iacovakis C, Athanasiou L, Birtsas P, et al.. (2011) Molecular detection and phylogenetic analysis of West Nile virus lineage 2 in sedentary wild birds (Eurasian magpie), Greece, 2010. Euro Surveill 16. 21586266

[21] Hijmans RJ, Cameron SE, Parra JL, Jones PG, Jarvis A (2005) Very high resolution interpolated climate surfaces for global land areas. International Journal of Climatology 25: 1965–1978.

[22] VanDerWal J, Shoo LP, Graham C, Williams SE (2009) Selecting pseudo-absence data for presence-only distribution modeling: How far should you stray from what you know? Ecological Modelling 220: 589–594.

[23] Warton D, Shepherd L (2010) Poisson point process models solve the “pseudo-absence problem” for presence-only data in ecology. The Annals of Applied Statistics 4: 1383–1402.

[24] Norusis M (2011) SPSS 19.0 Base. USA: SPSS Inc.

[25] Kaufman L, Rousseeuw PJ (1990) Finding Groups in Data: An Introduction to Cluster Analysis. New York: John Wiley & Sons.

[26] Hair JF, Black B, Babin B, Anderson R, Tatham R (2006) Multivariate Data Analysis (6^th^ Edition). Upper Saddle River, NJ: Prentice Hall.

[27] Ozdenerol E, Bialkowska-Jelinska E, Taff GN (2008) Locating suitable habitats for West Nile Virus-infected mosquitoes through association of environmental characteristics with infected mosquito locations: a case study in Shelby County, Tennessee. Int J Health Geogr 7: 12. 18373868 10.1186/1476-072X-7-12 PMC2322965

[28] Mostashari F, Bunning ML, Kitsutani PT, Singer DA, Nash D, et al (2001) Epidemic West Nile encephalitis, New York, 1999: results of a household-based seroepidemiological survey. Lancet 358: 261–264. 11498211 10.1016/S0140-6736(01)05480-0

[29] Bunning ML, Bowen RA, Cropp CB, Sullivan KG, Davis BS, et al (2002) Experimental infection of horses with West Nile virus. Emerg Infect Dis 8: 380–386. 11971771 10.3201/eid0804.010239 PMC3393377

[30] Komar N, Langevin S, Hinten S, Nemeth N, Edwards E, et al (2003) Experimental infection of North American birds with the New York 1999 strain of West Nile virus. Emerg Infect Dis 9: 311–322. 12643825 10.3201/eid0903.020628 PMC2958552

[31] Rappole JH, Derrickson SR, Hubalek Z (2000) Migratory birds and spread of West Nile virus in the Western Hemisphere. Emerg Infect Dis 6: 319–328. 10905964 10.3201/eid0604.000401 PMC2640881

[32] Malkinson M, Banet C, Weisman Y, Pokamunski S, King R, et al (2002) Introduction of West Nile virus in the Middle East by migrating white storks. Emerg Infect Dis 8: 392–397. 11971773 10.3201/eid0804.010217 PMC2730252

[33] Jourdain E, Zeller HG, Sabatier P, Murri S, Kayser Y, et al (2008) Prevalence of West Nile virus neutralizing antibodies in wild birds from the Camargue area, southern France. J Wildl Dis 44: 766–771. 18689669 10.7589/0090-3558-44.3.766

[34] Lopez G, Jimenez-Clavero MA, Tejedor CG, Soriguer R, Figuerola J (2008) Prevalence of West Nile virus neutralizing antibodies in Spain is related to the behavior of migratory birds. Vector Borne Zoonotic Dis 8: 615–621. 18399777 10.1089/vbz.2007.0200

[35] Papa A, Xanthopoulou K, Gewehr S, Mourelatos S (2011) Detection of West Nile virus lineage 2 in mosquitoes during a human outbreak in Greece. Clin Microbiol Infect 17: 1176–1180. 21781205 10.1111/j.1469-0691.2010.03438.x

[36] Bian L, Li L, Yan G (2006) Combining Global and Local Estimates for Spatial Distribution of Mosquito Larval Habitats. GIScience & Remote Sensing 43: 128–141.

[37] Patnaik JL, Juliusson L, Vogt RL (2007) Environmental predictors of human West Nile virus infections, Colorado. Emerg Infect Dis 13: 1788–1790. 18217573 10.3201/eid1311.070506 PMC3375805

